# A New Method for Re-Analyzing Evaluation Bias: Piecewise Growth Curve Modeling Reveals an Asymmetry in the Evaluation of Pro and Con Arguments

**DOI:** 10.1371/journal.pone.0148283

**Published:** 2016-02-03

**Authors:** Jens Jirschitzka, Joachim Kimmerle, Ulrike Cress

**Affiliations:** 1 Leibniz-Institut für Wissensmedien, Tübingen, Germany; 2 Department of Psychology, Eberhard Karls University of Tübingen, Tübingen, Germany; University of Geneva, SWITZERLAND

## Abstract

In four studies we tested a new methodological approach to the investigation of *evaluation bias*. The usage of *piecewise growth curve modeling* allowed for investigation into the impact of people’s attitudes on their persuasiveness ratings of pro- and con-arguments, measured over the whole range of the arguments’ polarity from an extreme con to an extreme pro position. Moreover, this method provided the opportunity to test specific hypotheses about the course of the evaluation bias within certain polarity ranges. We conducted two field studies with users of an existing online information portal (Studies 1a and 2a) as participants, and two Internet laboratory studies with mostly student participants (Studies 1b and 2b). In each of these studies we presented pro- and con-arguments, either for the topic of MOOCs (*massive open online courses*, Studies 1a and 1b) or for the topic of M-learning (*mobile learning*, Studies 2a and 2b). Our results indicate that using piecewise growth curve models is more appropriate than simpler approaches. An important finding of our studies was an asymmetry of the evaluation bias toward pro- or con-arguments: the evaluation bias appeared over the whole polarity range of pro-arguments and increased with more and more extreme polarity. This clear-cut result pattern appeared only on the pro-argument side. For the con-arguments, in contrast, the evaluation bias did not feature such a systematic picture.

## Introduction and Theoretical Background

At least since Leon Festinger’s “theory of cognitive dissonance” [1] and Sherif and Hovland’s (1961) “social judgment theory” [2] it is a well-known phenomenon that individuals’ prior attitudes and beliefs strongly influence how they deal with information and its sources. This is particularly the case if a controversial issue is highly relevant to the recipient and comes along with high affective involvement (e.g., [3–5]). Two kinds of consequences are of particular importance with regard to the impact of prior attitudes and beliefs: (a) selective seeking of attitude-consistent information while avoiding attitude-inconsistent information, and (b) overvaluing of attitude-consistent information while devaluing or even rejecting attitude-inconsistent information (e.g., [6]). While the most suitable term for the former consequences seems to be *selective exposure bias* (e.g., [7]), the most appropriate term for the latter seems to be *evaluation bias* (e.g., [8–9]).

Other common terms which refer in one sense or another to these phenomena of *motivated reasoning* [10] are: *biased assimilation* [2, 11–12], *boomerang/contrast effect*s [2, 13], *confirmation bias* [14–15], *congeniality bias* [3], *disconfirmation bias* [6, 16], *myside bias* [17–18], *partisan bias* [6, 19], and *prior attitude*/*belief effect* [6, 16]. For reasons of clarity, we will use the term *evaluation bias* here for referring to the influence of *attitudinal effects* on ratings of the arguments in which we were interested.

The attitudinal evaluation bias as well as the selective exposure bias are highly relevant for advertising and health prevention campaigns (e.g., [1, 20–21]), socio-political issues (e.g., [6, 22]), and determining media effects (e.g., [23–24]). In this sense, Druckman and Bolsen [22] concluded that “once individuals form initial opinions, they do not ‘objectively’ incorporate new factual information in ways often assumed by scientific literacy approaches” (p. 681). Moreover, in the current era of Web 2.0, selective exposure to attitude-consistent information and the devaluing of attitude-inconsistent information are frequently observable phenomena in online information searches, news consumption behavior, online forum discussions and voting on comments (e.g., [15, 25–27]).

In short, the more or less explicit underlying expectations of the corresponding studies were that people (a) search for attitude-consistent information and avoid attitude-inconsistent information and (b) evaluate attitude-consistent information considerably more favorably and accept it more frequently than attitude-inconsistent information. For example, Lord, Ross and Lepper [11] have shown that both proponents and opponents of the death penalty rated an attitude-supportive study (pro-attitudinal information) to be more convincing and more valid than an attitude-disconfirming study (con-attitudinal information). Another example is the also often cited work of Taber and Lodge [6], who found that pro-attitudinal arguments for affirmative action and gun control were rated as stronger than con-attitudinal information, regardless of whether the individuals’ prior attitudes were against or in favor of the measure in question.

In most of these studies, however, not only were the participants classified into proponent and opponent groups, but the arguments were also often dichotomized in pro- and con-arguments, even when it is intuitively obvious that attitudes as well as the degree of extremeness of pro- and con-arguments (their *polarity*) are more continuous rather than categorical variables. Such dichotomizing strategies may be appropriate in the early stages of research in a given field (e.g., [28]). But the dichotomizing of continuous variables (e.g., by median split) or comparisons of extreme groups (e.g., the lower against the upper quantile) is not only problematic from a statistical point of view [29–30], but also from a theoretical perspective. Such dichotomizing precludes important theoretical insights, as the following remarks illustrate.

**Fig 1:**
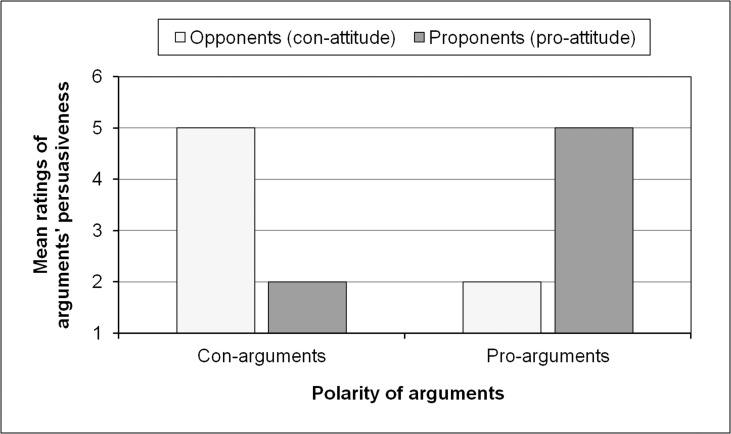
Typical assumption about the average ratings of the persuasiveness of con- and pro-arguments as a function of the prior attitudes of opponents and proponents.

For a hypothetical, controversial issue (e.g., death penalty), Fig 1 displays the typical assumption about the ratings of the *persuasiveness* of pro- and con-arguments as a function of the participants’ prior attitude. As Fig 1 indicates, pro-arguments (or con-arguments) are shown to be rated as more persuasive than con-arguments (or pro-arguments) by proponents (or opponents) and vice versa.

**Fig 2:**
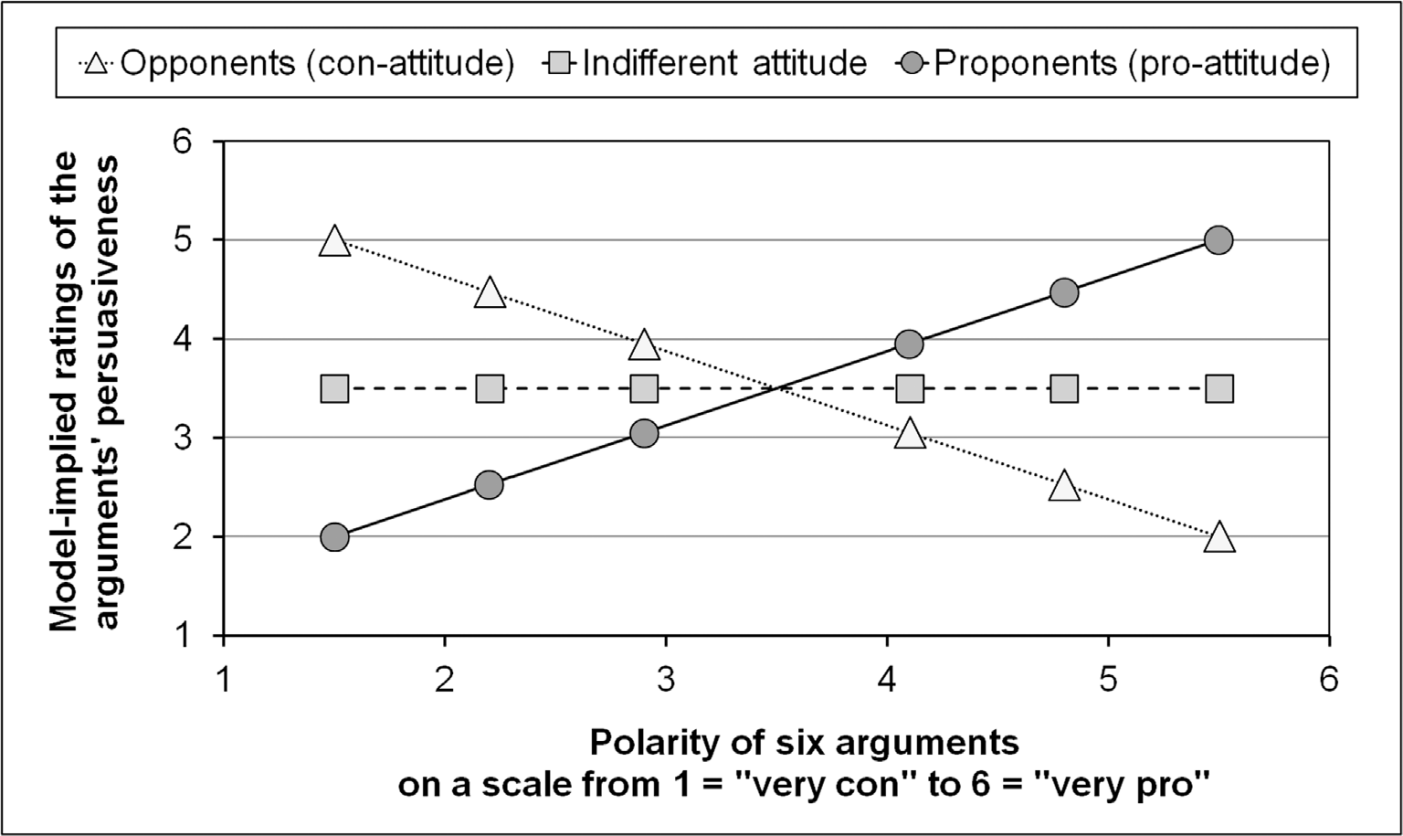
Hypothetical persuasiveness ratings if the continuous metric of arguments’ polarity is taken into account.

If both people’s prior attitudes and the polarity of arguments each are thought to have a continuous metric, however, the question occurs as to what the shapes of the resulting graphs would look like. A plausible answer could be that a pattern of line graphs would result as illustrated in Fig 2, (a) if the persuasiveness ratings are simple monotonic linear functions of the arguments’ polarity within each possible attitudinal level (within a range from extreme con to extreme pro), and (b) if the slope of these functions under these conditions is thought to be moderated by participants’ attitudes (i.e., an interaction between the predictor variables *polarity* and *attitude*). For purposes of illustration, we present the three-dimensionality of this hypothetical regression surface (polarity and attitude as predictor variables, and persuasiveness ratings as dependent variable). To do this we take three concrete values out of the range of possible values for attitude, although the underlying model should be specified and estimated with continuous variables [29]. In the hypothetical example in Fig 2, we show the persuasiveness ratings of six arguments with different *polarity scores*. As can be seen from Fig 2, the slope for the opponents should be negative (assigning their highest ratings to the most extreme con-arguments), whereas the slope of the proponents should be positive (assigning their highest ratings to the most extreme pro-arguments).

This idea has some significant shortcomings, however. First, the variables attitude and polarity are established on two different levels. And second, whereas attitude is a characteristic of individuals (the raters), polarity is a feature of the arguments. Nevertheless, the polarity score of an argument must also be extracted from human ratings, just as in attractiveness research, for example, where physical attractiveness of people must be extracted from the average of several individual ratings, and is treated as a “quasi-objective” characteristic of target persons (e.g., see [31]).

A possible methodological solution to this problem, which we have applied in the present analysis, is to use (*piecewise*) *growth curve modeling* that is (largely) equivalent to certain kinds of *hierarchic linear models* (HLM; e.g., see [32–37]). This approach allows for separate inspections of the polarity ranges of con- and pro-arguments, and thus can provide new insights for the research on evaluation bias.

The basic idea is that the persuasiveness ratings of *m* arguments can be specified as a repeated measure design with *m* time-points. An appropriate method for dealing with these kinds of data is *growth curve modeling* that allows for specifying linear and nonlinear trends over time whereby graphically the x-axis with the polarity variable represents a predictor continuum at a *within-level* (each individual rated the persuasiveness of several arguments with different polarity scores). For this within-level, growth curve models assume that each individual has his/her own growth curve with individual-specific regression parameters (e.g., individual intercepts and slopes for a linear regression of the persuasiveness ratings on polarity). As a consequence, each within-level regression parameter is a random variable with a mean and a variance. Further, on a *between-level*, such models allow for specifying these regression parameters as dependent variables (*intercepts- and slopes-as-outcome models*) [37], making it possible to study influences of some other variables (e.g., personal characteristics like attitude) on the shape of individual growth curves.

**Fig 3:**
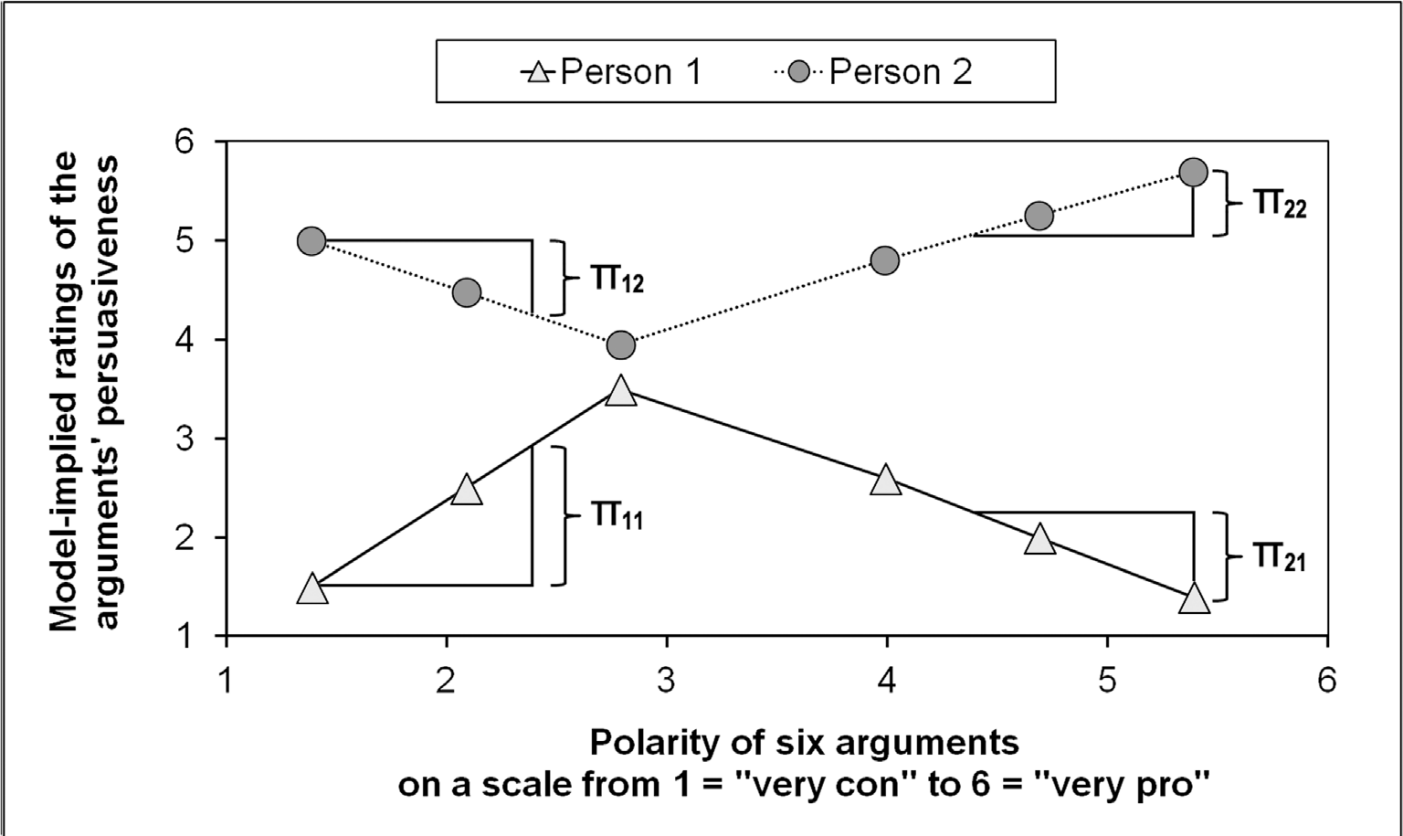
Hypothetical growth curves of two individuals. Individual slopes are labeled with π_11_, π_12_, π_21_, and π_22_.

In the present studies, we used *m* = 6 arguments (three con- and three pro-arguments), and therefore have six polarity scores on the x-axis (polarity as predictor on the within-level). This allows for splitting the linear growth curves into *two pieces* with different sizes and signs of the slopes describing separately the region of the three con- and the region of the three pro-arguments. So, if the slopes of both regions were in fact different and if the assumption of equal slopes (see Fig 2) does not hold, then such a model with different slopes for the two ranges would be more appropriate for describing individual trajectories. Fig 3 illustrates the idea of *piecewise growth curve models* (e.g., [32, 35, 37]) with two fictitious regression lines from two hypothetical individuals, with the ratings of the persuasiveness of six arguments as dependent variables. On the x-axis, the sequence of the arguments begins with the most extreme con-argument (the argument with the lowest polarity score) and ends with the most extreme pro-argument (the argument with the highest polarity score). As can be seen in Fig 3, hypothetical *person 1* has a positive within-level slope for the first three arguments (see Eq 1 below, whereupon this slope can be called π_11_), whereas the within-level slope of hypothetical *person 2* has a negative value (slope π_12_). The second within-slope for the last three arguments has a negative value for person 1 (slope π_21_) but a positive value for person 2 (slope π_22_).

Such a model with its bi-linear growth curves is mathematically represented in the four equations below [32, 35, 37]. Eq 1 specifies the model on the within-level for *i* = 1 to *n* individuals, whereby Y_ai_ is the persuasiveness rating from individual *i* of an argument with a polarity score *a*.

$$Y_{ai}=\pi_{0i}+\pi_{1i}\cdot \lambda_{1a}+\pi_{2i}\cdot \lambda_{2a}+\varepsilon_{ai}$$ (1)

The deviation of Y_ai_ from the individual model-implied growth trajectory is represented by the random effect ε_ai_ [35, 37]. The variables λ_1a_ and λ_2a_ are two coded variables that contain information about the polarity of an argument with the polarity value *a*. Thus, in such a piecewise regression model with two pieces, each polarity score *a* is represented by two values λ_1a_ and λ_2a_.

Different coding schemes for two-piece linear models can be found in [37]. In our study, the first scheme in ([37], p. 179) was applied. For illustration purposes, let us assume that there are six hypothetic polarity scores with the values of 1, 2, 3, 4, 5, and 6. The first step in this coding scheme is an additive transformation of the polarity scores, achieved by subtracting the first value from each value in order to set the first score to the value of zero. Thus, the resulting polarity scores would be 0, 1, 2, 3, 4, and 5. To represent these scores with the coding scheme described in [37], the parameter λ_2a_ for the pro-arguments would receive the value of zero for the first three con-arguments (λ_20_ = 0, λ_21_ = 0, and λ_22_ = 0), and therefore would not play a role for the three con arguments. The parameter λ_1a_ would receive the first three (difference-transformed) polarity scores for its first three values (λ_10_ = 0, λ_11_ = 1, and λ_12_ = 2), would be fixed at the third polarity score for its last three values (λ_13_ = 2, λ_14_ = 2, and λ_15_ = 2), and therefore would not play a role for the three pro arguments. The parameter λ_2a_ would receive the differences between the last three polarity scores (3, 4, and 5) and the third polarity score (2) for its last three values (λ_23_ = 1, λ_24_ = 2, and λ_25_ = 3). So in our example, the values for λ_1a_ would be 0, 1, 2, 2, 2, and 2, and the values for λ_2a_ would be 0, 0, 0, 1, 2, and 3. If we insert these values into Eq 1 to estimate Y_ai_ for each of the six polarity scores, we would obtain for the first three polarity scores: π_0i_, π_0i_ + π_1i_, and π_0i_ + π_1i_·2. For the last three polarity scores we would obtain: π_0i_ + π_1i_·2 + π_2i_, π_0i_ + π_1i_·2 + π_2i_·2, and π_0i_ + π_1i_·2 + π_2i_·3 (see [37], p. 178–179).

As a consequence of this coding scheme, the parameter π_1i_ is the individual slope for the con-arguments, and π_2i_ is the individual slope for the pro-arguments. The intercept π_0i_ can be interpreted as an expected persuasiveness rating, if both λ_1a_ and λ_2a_ take a value of zero (with the coding scheme applied here, this holds for the most extreme con-argument), and if the predictor variable (attitude) on the between-level also takes a value of zero (see Eqs 2–4). If this value has no practical meaning, it is necessary to center the predictor before the analysis (e.g., see [29]).

Since π_0i_, π_1i_, and π_2i_ are random variables that vary among individuals, they can be explained by another person variable Z (e.g., attitude) on the between-level. This is expressed in Eqs 2–4, whereby the intercepts β_00_, β_10_, and β_20_ as well as the slopes β_01_, β_11_, and β_21_·in these formulas are fixed effects, while the individual deviations ζ_0i_, ζ_1i_, and ζ_2i_ of the individual growth parameters from the predicted growth parameters are random effects [35, 37].

$$\pi_{0i}=\beta_{00}+\beta_{01}\cdot Z_{i}+\zeta_{0i}$$ (2)

$$\pi_{1i}=\beta_{10}+\beta_{11}\cdot Z_{i}+\zeta_{1i}$$ (3)

$$\pi_{2i}=\beta_{20}+\beta_{21}\cdot Z_{i}+\zeta_{2i}$$ (4)

Eq 5 results from inserting Eqs 2–4 into Eq 1 and restructuring accordingly. In Eq 5, the term (β_01_ + β_11_·λ_1a_ + β_21_·λ_2a_) represents the effect of predictor Z on the persuasiveness ratings for polarity score *a*. With regard to attitude as predictor Z, this term and its values represent a measure for the size and direction of an *attitudinal evaluation bias* at a certain polarity score *a*.

$$Y_{ai}=(\beta_{00}+\beta_{10}\cdot \lambda_{1a}+\beta_{20}\cdot \lambda_{2a})+(\beta_{01}+\beta_{11}\cdot \lambda_{1a}+\beta_{21}\cdot \lambda_{2a})\cdot Z_{i}+(\zeta_{0i}+\zeta_{1i}\cdot \lambda_{1a}+\zeta_{2i}\cdot \lambda_{2a}+\varepsilon_{ia})$$ (5)

Important assumptions that go along with Eqs 1–4 are (a) that on the within-level, each individual regression of the persuasiveness ratings on the first three argument scores as well as the regression on the last three argument scores are linear and (b) that on the between-level, the regressions of the intercept π_0i_ and the slopes π_1i_ and π_2i_ on the predictor Z are also linear. Theoretically, with enough arguments and their scores, it would also be possible to specify polynomial models on the within-level to fit individual growth curves and to specify higher order regressions for growth parameters on the between-level [37]. However, the corresponding results are harder to construe, as Bollen and Curran [32] point out: “higher-order polynomial trajectory models become increasingly difficult to interpret when relating model results back to theory” (p. 97). Additionally, it seems intuitively plausible to use a linear piecewise model that splits the whole regression line into two pieces: one for the con-arguments and another piece for the pro-arguments. Moreover, such a piecewise model with two slopes is already much more flexible than the linear growth curve model with only one slope which is so often used.

## Hypotheses

From the remarks above and especially from the theoretical and methodical considerations that are presented graphically in Figs 2 and 3, we derive the following hypotheses about the influence of *attitude* on the evaluation of the *persuasiveness* of pro- and con-arguments with different degrees of extremeness (*polarity*):

### Hypothesis H-1

Individual trajectories (the attitude-dependent course of individual persuasiveness ratings over the whole polarity range) should be better represented with a piecewise (bi-linear) growth curve model with two different within-level slopes (π_1i_ for con-arguments and π_2i_ for pro-arguments in Eq 1; see Fig 3) than with a model that has only one slope (see Fig 2). That means that the within-level slopes π_1i_ and π_2i_ should not be the same (as in Fig 3). Therefore, either the between-level intercepts β_10_ and β_20_, or the between-level slopes β_11_ and β_21_ (Eqs 3 and 4), or both should not have the same values (β_10_ and β_20_ are unequal and/or β_11_ and β_21_ are unequal).

### Hypothesis H-2

The evaluation bias should show negative values for all three con-arguments (H-2a for the most extreme con-argument “—-”, H-2b for the moderately extreme con-argument “—” and H-2c for the lowest extreme con-argument “-”) and positive values for all three pro-arguments (H-2d for the most extreme pro-argument “+”, H-2e for the moderately extreme pro-argument “++” and H-2f for the lowest extreme pro-argument “+++”). As an influence of attitude on the persuasiveness ratings for any given argument, the evaluation bias for that given argument is represented by the value of the term (β_01_ + β_11_·λ_1a_ + β_21_·λ_2a_) in Eq 5.

### Hypothesis H-3

For con-arguments, the evaluation bias should be strongest for the most extreme con-argument and lowest for the lowest extreme con-argument. That is, the evaluation bias of the most extreme con-argument should be higher (in absolute value) than the bias of the moderately extreme con-argument (H-3a) and higher than the bias of the lowest extreme con-argument (H-3b). The evaluation bias of the moderately extreme con-argument should be higher than the bias of the lowest extreme con-argument (H-3c). An analogous pattern should hold for the pro-arguments. The evaluation bias of the most extreme pro-argument should be higher than the bias of the moderately extreme pro-argument (H-3d) and higher than the bias of the lowest extreme pro-argument (H-3e). The evaluation bias of the moderately extreme pro-argument should be higher than the bias of the lowest extreme pro-argument (H-3f).

Further, and in a cross-validating sense, the hypotheses above should be valid for *different topics*. Additionally, the expected result patterns should hold for different subgroups of people who may have different perspectives on the topics in question.

## Materials and Methods

With two topics, MOOCs (*massive open online courses*, Study 1) and M-learning (*mobile learning*, Study 2), we ran two studies, each considering two different participant groups. Participants in the first group were regular users who navigated an existing web information portal and came across the presented material in field studies (Studies 1a and 2a). Participants from the second group were mostly university students who were invited to participate in online studies in order to navigate the same material (Studies 1b and 2b). So the first group represents an existing, ecological valid sample of information searchers on the Web, whereas the second sample encountered the information in a more controlled online laboratory setting.

### Participants

The study material was embedded as HTML iframe parts into the website *e-teaching*.*org* (www.e-teaching.org). This website is an Internet portal which has been offered and hosted by our institute for years, providing information about teaching with digital media. As a well-established and award-winning portal, it is well-known in the German-speaking e-teaching community as a place to get relevant and up-to-date information about e-teaching and e-learning, as well as to network with other professionals or organizations. The portal mainly addresses lecturers who use digital media for teaching in higher education. Because the portal in 2014 featured specials about MOOCs and M-learning, we used these topics as study material in two consecutive studies (Study 1a and Study 2a). Both topics are controversially discussed. So we could expect them to cause the kind of evaluation biases we aimed to analyze. For the sample of portal users we integrated an informed consent form into the website. When users came across the relevant iframe they were informed that we would use these pages for scientific analysis purposes and that they could leave these sites whenever they wanted to.

We recruited the second group of participants (Studies 1b and 2b) with an online recruitment system that is regularly used in our institute to invite people (primarily university students) to be participants in empirical studies. In the invitation e-mail we did not tell them about the specific content or nature of the study. These participants had to navigate the same portal-like pages with the same material used in Studies 1a and 2a. Thus, Study 1b provided information about MOOCs and Study 2b dealt with information about M-learning. As a compensation for their participation, participants in Studies 1b and 2b could enter a lottery where 10 participants had the opportunity to win 20 Euros each.

In addition to the participants described below, some individuals with missing data on all six dependent variables (see below) were not included in the following analyses. From initially *n* = 545 participants who visited at least the first page with informed consent content, the sample which remained contains *n* = 349 individuals who visited the online questionnaire *and* rated at least one of the six arguments (one participant had to be excluded because s/he wished to withdraw her/his data). The data file for the main analyses is given in S1 File.

Given the sample of portal users and the natural setting of their participation, however, we cannot be sure whether these people constituted two entirely disjunctive samples in Study 1a and Study 2a (it is possible that some individuals participated in both studies). The samples in Study 1b and Study 2b were entirely disjunctive, however. Table 1 summarizes information about sample size, sex ratio, domain-specific knowledge, attitude, and some other characteristics of the four groups of participants.

**Table 1:** Sample description: Participants who rated at least one argument.

Sample characteristics	Topic: MOOCs (Study 1)		Topic: M-learning (Study 2)	
	Study 1a	Study 1b	Study 2a	Study 2b
Total: *n*	69	110	60	110
Female: *n* (%)	27 (39.1%)	80 (72.7%)	25 (41.7%)	77 (70.0%)
Male: *n* (%)	26 (37.7%)	22 (20.0%)	21 (35.0%)	27 (24.5%)
No sex information: *n* (%)	16 (23.2%)	8 (7.3%)	14 (23.3%)	6 (5.5%)
Domain-specific, self-rated knowledge on a 6-point scale: *M* (*SD*)	4.55 (1.19)	1.86 (0.96)	3.89 (1.19)	2.45 (1.10)
Information (knowledge) obtained from: *n* (%)	67 (97.1%)	109 (99.1%)	59 (98.3%)	109 (99.1%)
Topic-related (pro-)attitude on a 6-point scale: *M* (*SD*)	3.81 (1.27)	3.50 (1.01)	4.76 (1.02)	3.65 (1.14)
Information (attitude) obtained from: *n* (%)	66 (95.7%)	107 (97.3%)	59 (98.3%)	109 (99.1%)
Age: *M* (*SD*)	40.12 (8.86)	22.47 (4.42)	41.49 (10.70)	22.92 (4.37)
Information (age) obtained from: *n* (%)	52 (75.4%)	102 (92.7%)	43 (71.7%)	105 (95.5%)
Age range	23 to 58	18 to 54	24 to 62	18 to 42
Employee status	Mostly employed in educational settings	Mostly students	Mostly employed in educational settings	Mostly students
Recruitment context	Online portal e-teaching.org	Online recruitment system	Online portal e-teaching.org	Online recruitment system

Regarding the employee-status, 50 (72.5%) participants in Study 1a and 46 (76.7%) participants in Study 2a belonged to at least one of the following groups: middle or high school teachers, college lecturers, lecturers in continuing or adult education, researchers in academic and non-academic fields, other employees in university institutions, employees in business companies, and self-employed individuals. Ninety-three (84.5%) participants in Study 1b and 100 (90.9%) participants in Study 2b indicated that they were university students.

### Material and Pilot Studies

For the topic of MOOCs (Study 1) as well as for the topic of M-learning (Study 2), six arguments were presented on the computer screen with six corresponding buttons, arranged in a horizontal line, and labeled in German language with “open the argument”. Below these buttons was a line with minuses and pluses standing for the polarity of the argument: “—-” (most extreme con-argument), “—”, “-”, “+”, “++”, “+++” (most extreme pro-argument). At its ends, the scale was also labeled with the words “contra” and “pro”. The buttons for the con-arguments and the corresponding region of the line below them were colored in red and the pro-arguments as well as the region of the line below them were colored in green. We randomly assigned the placement of the presented arguments on the screen (“—-” to “+++” from left to right or from right to left) in order to control for order effects. We also randomly varied the degree of the intensity of the color: in a dichotomized manner or in a more continuous rainbow-like manner. For the focus here and the corresponding variables of interest, these efforts were not relevant, however, even with regard to potential effects and interactions, which were not found to be substantial.

The M-learning arguments were constructed from discussion points which were typical for this topic, whereas the MOOC arguments were taken mainly from a position paper on MOOCs from the German Rectors’ Conference [38]. Each argument was in the German language and consisted of either 43 words (MOOC arguments in Studies 1a and 1b) or 44 words (M-learning arguments in Studies 2a and 2b). In order to select appropriate arguments, we conducted two pilot studies where experts (*n* = 7 for MOOCs and *n* = 9 for M-learning) rated the polarity and persuasiveness of 37 MOOC and 24 M-learning arguments using six-point rating scales. Inter-rater agreement was estimated with the one-way random single measures intraclass correlation ICC(1,1) [39] (for a missing-tolerant approach, see [40]) and can be considered to be good, ICC(1,1) = .68 for MOOC arguments and ICC(1,1) = .64 for M-learning arguments (for the corresponding classification, see [41]). For the final experiments we chose six arguments such that each (a) could be ordered on a con-pro continuum, for which (b) the corresponding inter-rater reliability was comparatively high (indicated by the variance and the agreement score per argument; e.g., see [42]), and for which (c) the persuasiveness scores were on approximately the same level. We also took care to choose arguments which were suitable with regard to content for presenting them in the Internet portal we used. The polarity scores (average over the expert raters; e.g., see [43]) of the six selected MOOC arguments were: 1.86, 2.33, 3.17, 4.57, 4.86, and 5.29. Therefore, after applying the coding scheme for piecewise regression models as described in [37] and outlined above, the resulting values for λ_1a_ were 0.00, 0.48, 1.31, 1.31, 1.31, and 1.31 and the resulting values for λ_2a_ were 0.00, 0.00, 0.00, 1.40, 1.69, and 2.12 (see S2 File for more decimal places). The polarity scores of the six selected M-learning arguments were: 2.11, 2.33, 2.75, 4.67, 5.00, and 5.33. The resulting values for λ_1a_, after applying the coding scheme, were 0.00, 0.22, 0.64, 0.64, 0.64, and 0.64 and the resulting values for λ_2a_ were 0.00, 0.00, 0.00, 1.92, 2.25, and 2.58.

Here is an example for the most extreme con-MOOC argument and an example for the most extreme pro-MOOC argument:

“MOOCs increase the trend toward shorter educational formats. Their dangers are that education will be more fragmented, that larger contexts will no longer be teachable, and that students will no longer be required to read, to understand, or to transfer complex and comprehensive material.” (con-argument—).

“Interdisciplinarity and transdisciplinarity are often outspoken ideal wishes for research projects and courses, but these wishes are realized to a lesser extent than is desired. MOOCs can fulfill these claims. Moreover, they can contribute to extending the range of the lecture series’ classical university format on a global scale.” (pro-argument +++).

### Control Variables, Predictor, and Dependent Variables

At the beginning of our studies, we asked the participants about their *self-rated knowledge* about MOOCs or M-learning respectively (e.g., “I would guess that my knowledge about MOOCs is relatively high”) with three six-point Likert-scale items (1 = *not at all true*, 6 = *completely true*), whereby one item was inversely worded. The internal consistency (Cronbach’s alpha) for this scale was α = .87 (MOOCs) and α = .76 (M-learning) respectively. The *attitude* about the topic was also measured with three six-point Likert-scale items (e.g., “MOOCs should become an important part of university education”, 1 = *not at all true* 6 = *completely true*), whereby one item was inversely worded. The internal consistency (Cronbach’s alpha) for this scale was α = .77 (MOOCs) and α = .87 (M-learning) respectively.

The dependent variable was the perceived persuasiveness for each of the six arguments. Each argument was presented together with a six-point one-item rating scale (1 = *not at all convincing*, 6 = *very convincing*). If an argument was opened and was rated more than once, the evaluations of this participant were averaged for this argument. We also tracked the navigation behavior of the users to control for the order in which the arguments were opened.

### Procedure

Our studies were conducted with approval from the institutional ethics committee of the Leibniz-Institut für Wissensmedien (Tübingen, Germany; approval numbers: LEK 2014/022, LEK 2014/023, LEK 2014/037, LEK 2014/038, and LEK 2014/039). Participants gave their written informed consent. After that, a website opened asking for participants’ topic-relevant knowledge about and attitude toward MOOCs or M-learning respectively. Then, six buttons to open each of the six arguments separately appeared on the screen. The buttons could be activated without any coercion to begin with a particular argument. Altogether, the buttons could be clicked a total of seven times, regardless of which button had been opened previously (so an argument could be opened twice or more). There was also the option not to open any argument. After participants clicked on a button, the corresponding argument appeared. It is worthy to note that with 299 out of 349 participants (85.7%), a great majority began reading the argument on the left-hand side, regardless of whether the first argument was the most extreme con- or the most extreme pro-argument. This was probably due to the fact that the customary reading direction is from left to right. It was possible for participants to read every argument and rate its perceived persuasiveness. Whether an argument was rated or not, the text could be closed and another text could be opened, or the same text could be opened again.

Whenever a participant wanted to exit or after she had clicked the maximum of seven buttons, a new questionnaire-like page opened and the attitude items appeared at the beginning. Some other topic-related items followed as well (e.g., about MOOCs or the ownership of mobile devices), which are not relevant here. Additionally, each participant had the opportunity to write some statements about the topic or the questionnaire in general. At the end of the questionnaire, demographics questions were asked (age, sex, and employee-status). Participants in Study 1b and 2b had the opportunity to become informed about the purposes of the study via e-mail. For the participants in Studies 1a and 2a (users of e-teaching.org), some descriptive results of the study (e.g., ratings of the arguments) were introduced on the website and via newsletters.

### Data Analysis Methodology

Estimates of the piecewise growth curve models were done with Mplus 7.3 [44]. The Mplus syntax is given in S2 File. Instead of using the Maximum Likelihood estimator, we applied *Bayesian estimation* [45–47]. Among others, important advantages of Bayesian estimation are (a) that it can be used even with small sample sizes, (b) non-normality can be handled better, and (c) estimations of implausible values (e.g., negative variances) are impossible [46–47]. Missing data were appropriately dealt with by the Bayes full-information estimator under the assumption of MAR (missing at random) [48–49].

In the following Bayesian growth curve analyses, non-informative priors were used for the Bayesian estimation procedure and the medians of the posterior distributions were used as point estimates [46–47]. The posterior distributions for the parameters were estimated with the Markov chain Monte Carlo (MCMC) Gibbs sampler algorithm (e.g., see [46, 50–51]). For each model, two MCMC chains were used. The convergence criterion was repeatedly assessed (each time after 100 iterations; e.g., see [50]) on the basis of the final half of all iterations per chain. If the criterion was reached, the first half of all iterations from both chains were dropped (burn-in phase) and the posterior distributions were built from the remaining post-burn-in iterations (e.g., see [44, 50]). Taking the burn-in phase and the post-burn-in phase together, we specified 30,000 as a minimum and 200,000 as a maximum for the total number of iterations per chain.

For determining the convergence, we used the Gelman-Rubin convergence criterion [44, 52]. Convergence is reached when the Potential scale reduction (PSR; see [53]) is smaller than: a = 1 + b · c, where c is 2 for a large number of parameters in the model (see [44], p. 634). Thus, because we set b to the value of 0.001, the PSR had to be smaller than 1.002. This is a very strict criterion, since the PSR should be very close to 1.00, and values of 1.05 already indicate a good convergence (e.g., see [46–47, 50]).

With Bayesian estimation, credibility intervals (not necessesarily symmetric) are produced for estimated parameters about which statements can be made; for instance, that there is “a 95% probability that the population value is within the limits of the interval”([46], p. 844, see also [54–56]). If the value zero does not lie within this interval, the result can be interpreted as significant according to classical frequentist null hypothesis testing [46]. However, the essential focus of Bayesian analyses is not on conditional probabilities of the data given certain (null) hypotheses, as in the frequentist approach, but on posterior conditional probabilities of hypotheses given the data (e.g., see [54–56]). Although this is the case, we still address the multiple testing problem, that is, the overall risk of false significance alarms which increases with the number of conducted significance tests (e.g., see [57–58]), by using more conservative 99% credibility intervals instead of 95% credibility intervals for the posterior distributions. Model comparisons (e.g., a model with parameter constraints vs. a model with freely estimated parameters) were made with the *deviance information criterion* (DIC; [59]), whereby from two competing models the model with the smaller DIC should be chosen as the better model (e.g., see [51, 60–61]).

## Results

First, we will present the findings regarding the piecewise growth curve models that were estimated. Then we will provide the results of the hypothesis testing.

### Piecewise Growth Curve Models

The following analyses consisted of two multi-group piecewise growth curve models with freely estimated within-level and between-level parameters: (a) one separate multi-group model for the MOOC topic and (b) one multi-group model for the topic of M-learning. The measured attitude toward the corresponding topic served as between-level predictor. For model estimation, the polarity value for the most extreme con-argument was fixed to the value of zero, which implies a difference transformation of the polarity values of all six arguments. The attitude variable was centered at the theoretical midpoint of the scale (3.5).

The first model (MOOC topic) converged after 71,700 iterations (PSR < 1.002). Therefore, the final 35,850 iterations from each of the two chains were used to build the posterior distributions (post-burn-in phase). The second model (M-learning) converged after 40,400 iterations (PSR < 1.002), so the final 20,200 iterations from each of the two chains were used to build the posterior distributions.

The group-specific between-level parameters for the prediction of the within-level parameters from attitude are displayed in S1, S2, and S3 Tables. The significance of the parameters regarding the value of zero can be concluded from the Bayesian 99% credibility interval for each parameter. If an interval does not contain the value of zero, the estimated parameter can be regarded as significant.

**Fig 4:**
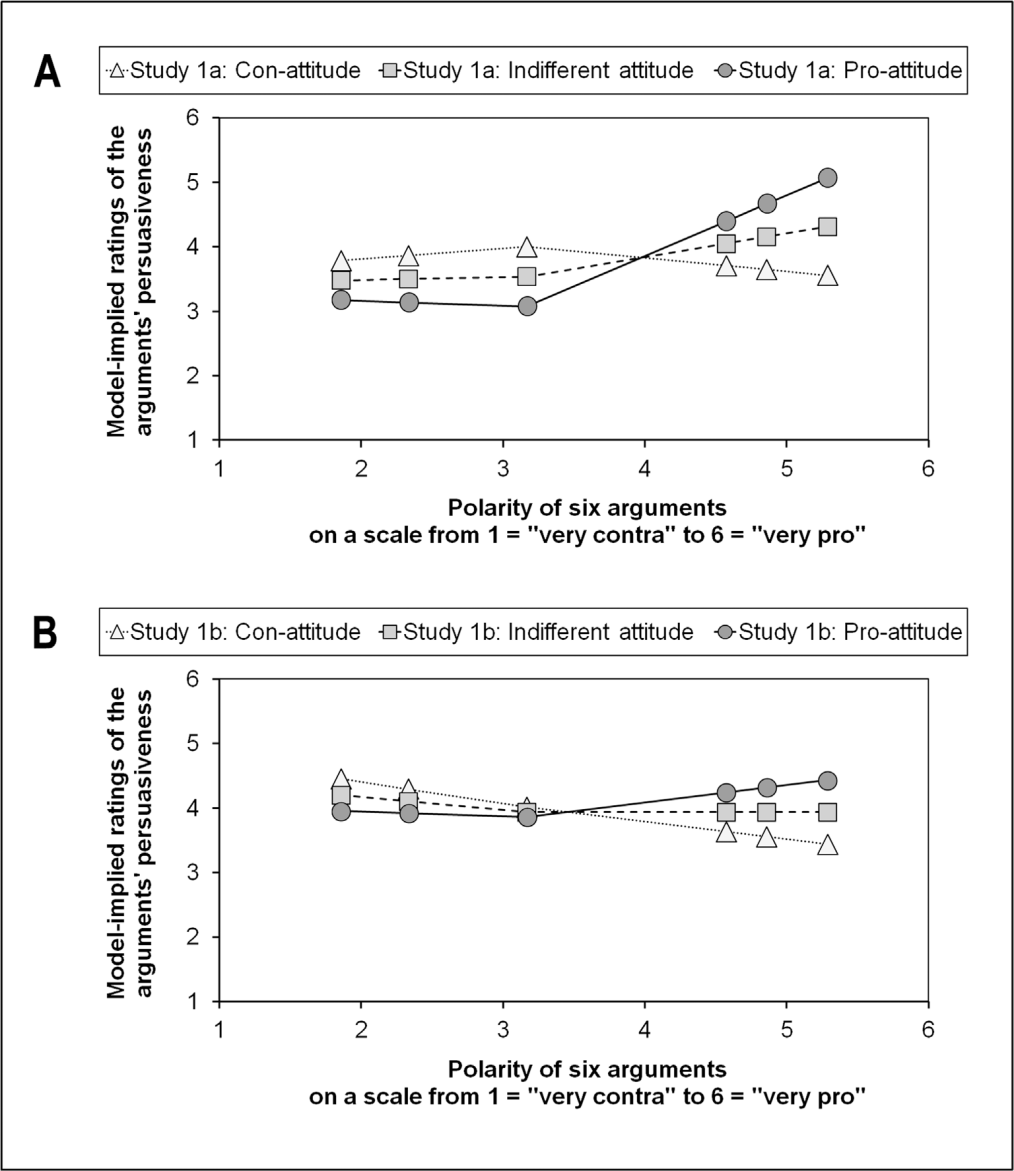
Piecewise growth curves: MOOCs. (A) Study 1a. (B) Study 1b.

**Fig 5:**
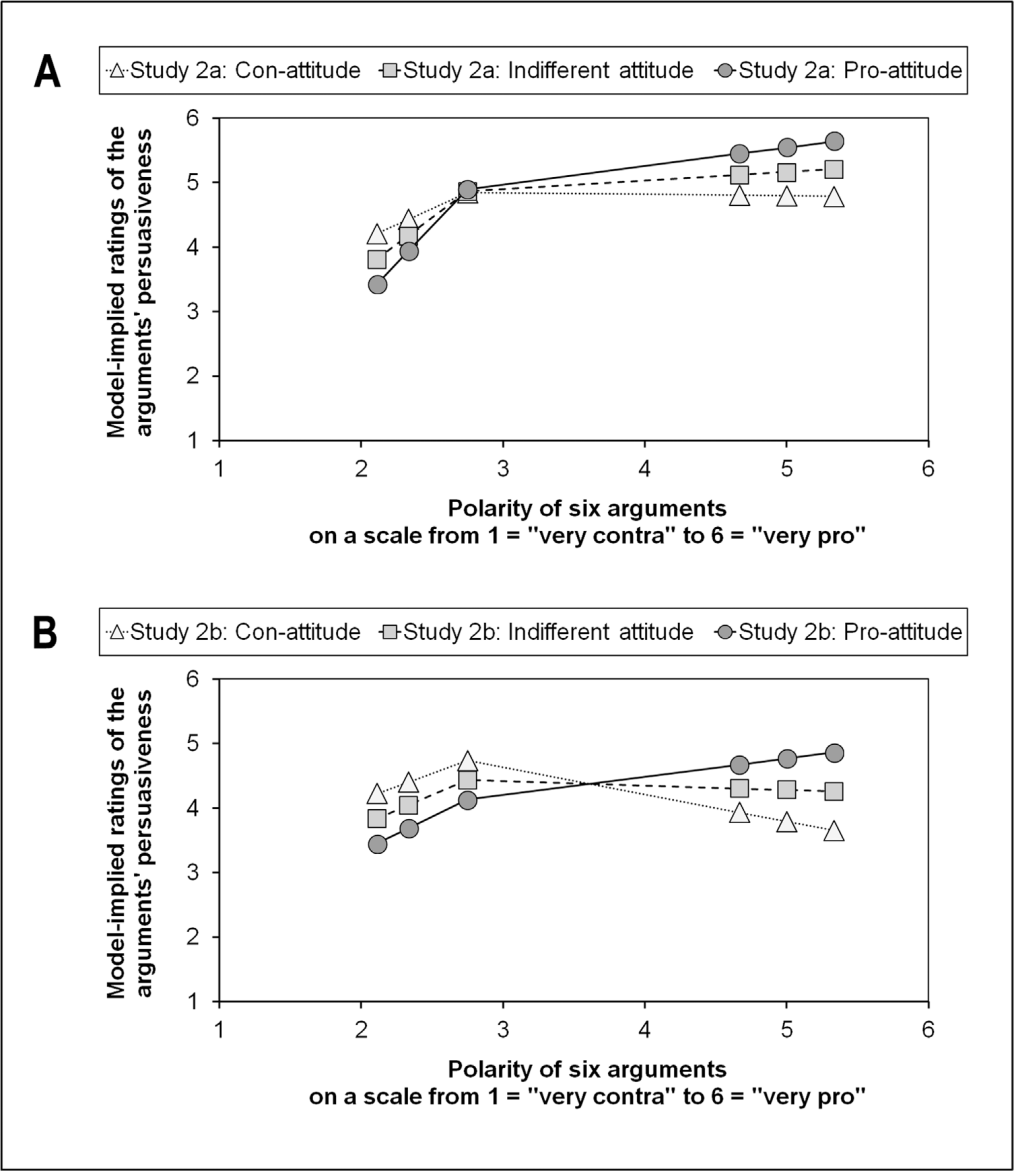
Piecewise growth curves: M-learning. (A) Study 2a. (B) Study 2b.

To illustrate the results graphically, for each topic and separately for each group of participants, within-level parameters (π_0i_, π_1i_, π_2i_) of the growth curves within each group were predicted (a) from attitude values that were one group-specific standard deviation below the group mean of the midpoint-centered attitude variable, (b) from values that resembled the attitude group mean of the midpoint-centered attitude variable, and (c) from values that were one group-specific standard deviation above the group mean. Fig 4 shows these curves for the MOOC topic (Studies 1a and 1b) and Fig 5 for the M-learning topic (Studies 2a and 2b). For purposes of simplification with regard to Figs 4 and 5, the polarity values of the arguments on the x-axis were back-transformed to the original values which were taken from the two pilot studies.

As most of the graphs in Figs 4 and 5 indicate, only the slopes for the pro-arguments were influenced by attitude, whereby stronger pro-attitudes came along with comparably steeper positive slopes for the individual trajectories at the polarity-range of the pro-arguments. At the same time, con-attitudes went hand in hand with negative slopes. In other words, increases in the extremeness of pro-arguments led to increases of differences in the persuasiveness ratings between individuals with pro-attitudes and individuals with con-attitudes. This phenomenon is asymmetrical, as it seems to be the case only with pro-arguments. For con-arguments, potential attitude-dependent differences in the persuasiveness ratings remained rather stable, regardless of the extremeness of the con-arguments.

This result pattern was also represented in the size and significance of the between-level slopes (see S2 and S3 Tables). For the prediction of the within-level slope for the con-arguments (π_1i_), there was no significant effect of attitude (see S2 Table). With regard to the within-level slope for the pro-arguments (π_2i_), however, in three of the four groups we found a significant positive effect of attitude (see S3 Table). This means that the shape of the first part of the individual piecewise trajectories (the persuasiveness ratings of the three con-arguments) was not affected by the attitude toward the topic. However, the shape of the second part of the individual piecewise trajectories (the persuasiveness ratings of the three pro-arguments) was affected by the attitude in such a way that the stronger the attitude, the higher was the slope of this second part of the trajectories; and the weaker the attitude, the lower (or more negative) was the slope of this second piece. Even though there was no significant effect of attitude on π_2i_ in the M-learning group of portal users (Study 2a), it is remarkable that also this parameter has a positive sign (see S3 Table).

### Hypothesis H-1

To be able to determine whether using a (bi-linear) piecewise growth curve model was more appropriate than just using a linear growth curve model with only one single within-level slope for the whole polarity range from con to pro (as in Fig 2), both models were compared in each study with the help of the *deviance information criterion* (DIC). The model with the smaller DIC would be regarded as the better model. The simple linear growth curve models had the specification that the between-level intercepts β_10_ and β_20_ as well as the between-level slopes β_11_ and β_21_ were held equal. The results for all four studies are shown in Table 2.

**Table 2:** Model comparisons (model with parameter constraints: β_10_ and β_20_ as well as β_11_ and β_21_ are held equal) with the deviance information criterion (DIC).

Topic	Group	*n*	DIC for the piecewise model with parameter constraints	DIC for the piecewise model without parameter constraints	Smaller DIC speaks for the model:
MOOCs	Study 1a	69	3947.24	3945.84	without constraints
	Study 1b	110	3942.92	3945.84	with constraints
M-learning	Study 2a	60	3767.78	3758.59	without constraints
	Study 2b	110	3763.83	3758.59	without constraints

As can be concluded from Table 2, for three of the four studies, a (bi-linear) piecewise model was shown to be to be more appropriate than a simple growth curve model with only one slope. Thus, three of the four studies support H-1.

### Hypothesis H-2

**Fig 6:**
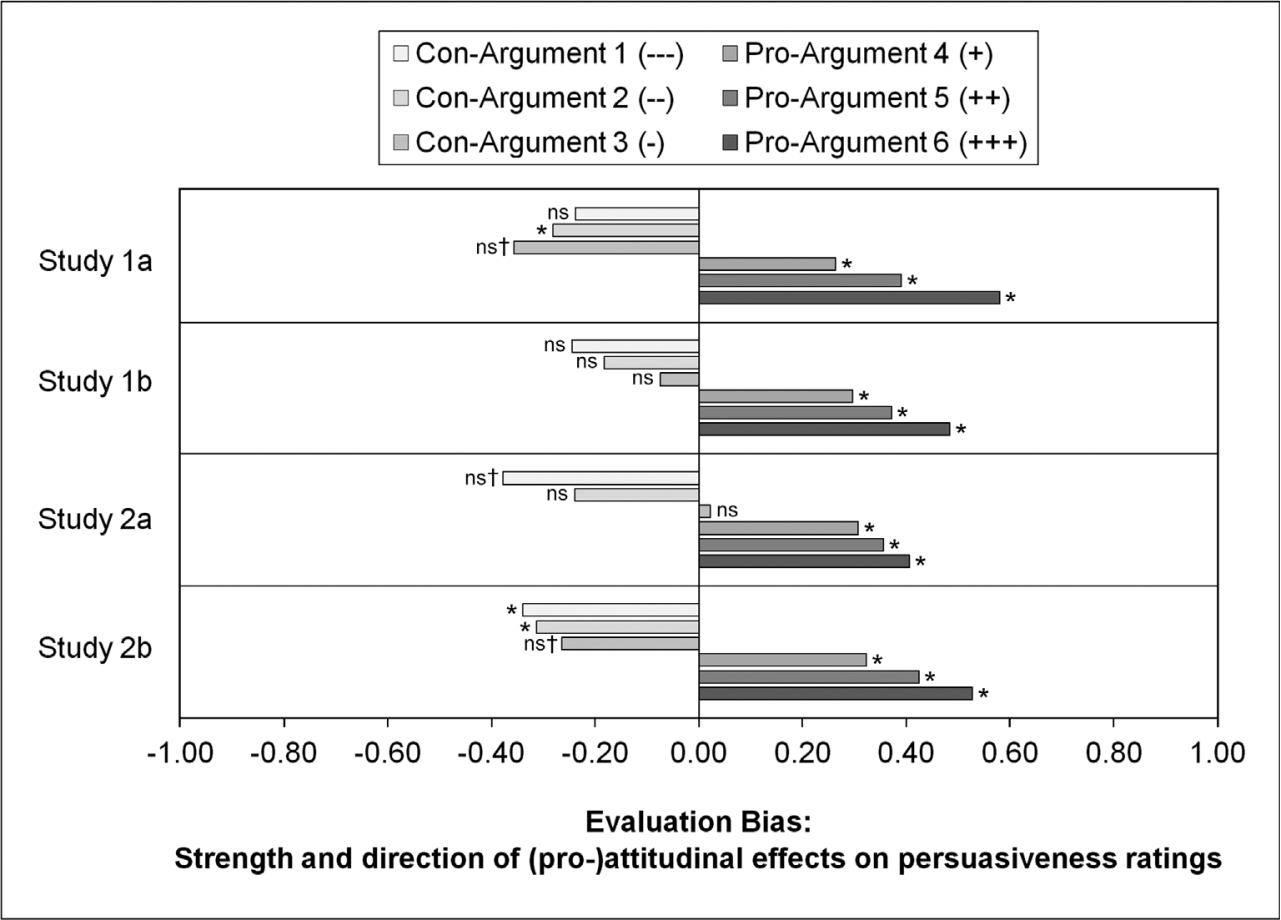
Attitudinal evaluation bias: Sign, size, and significance of the influence of attitude on the persuasiveness ratings for each argument for each of the four studies. (*) Bayesian 99% credibility interval for evaluation bias does not contain the value of zero (significant). (ns) Bayesian 99% credibility interval contains the value of zero (not significant). (†) A 95% credibility interval would not contain the value of zero.

The sign, size, and significance of the influence of attitude on the persuasiveness ratings (*attitudinal evaluation bias*; see Eq 5) for each argument are depicted in Fig 6 for all four studies. The corresponding numerical results are given in S4 and S5 Tables.

On the basis of this result pattern, we conclude that hypotheses H-2d (for argument “+”), H-2e (“++”) and H-2f (“+++”) for significant and positive evaluation biases toward the *pro-arguments* can be maintained within all four studies. For the con-arguments, hypothesis H-2a (“—-”) can be maintained only in Study 2b and must be rejected for Studies 1a, 1b, and 2a. Hypothesis H-2b (“—”) can be maintained only in Studies 1a and 2b and must be rejected for Studies 1b and 2a. Hypothesis H-2c (“-”) must be rejected within all four studies.

### Hypothesis H-3

In the final step, we compared evaluation biases of all three con- and all three pro-arguments with each other, for each kind of argument. Table 3 shows the results of these comparisons for the con-arguments. As Table 3 indicates, for the con-arguments the postulated pairwise differences with regard to the evaluation bias magnitude were not significant in all four studies. That is, there were no significant differences and seemingly no covariation of the evaluation bias with the polarity extremeness of the con-arguments.

**Table 3:** Pairwise comparisons between attitudinal evaluation biases within the con-arguments. ns: Bayesian 99% credibility interval contains the value of zero (not significant).

Group	*n*	Comparison between evaluation biases for	Estimated difference	Bayesian 99% credibility interval [lower 0.5%, upper 0.5%]	Significance
Study 1a	69	(---) vs. (—)	0.04	[-0.18, 0.27]	ns
Study 1b	110	(---) vs. (—)	-0.06	[-0.24, 0.12]	ns
Study 2a	60	(---) vs. (—)	-0.14	[-0.37, 0.09]	ns
Study 2b	110	(---) vs. (—)	-0.03	[-0.18, 0.13]	ns
Study 1a	69	(---) vs. (-)	0.12	[-0.49, 0.75]	ns
Study 1b	110	(---) vs. (-)	-0.17	[-0.66, 0.32]	ns
Study 2a	60	(---) vs. (-)	-0.40	[-1.08, 0.27]	ns
Study 2b	110	(---) vs. (-)	-0.08	[-0.53, 0.37]	ns
Study 1a	69	(—) vs. (-)	0.08	[-0.31, 0.48]	ns
Study 1b	110	(—) vs. (-)	-0.11	[-0.42, 0.20]	ns
Study 2a	60	(—) vs. (-)	-0.26	[-0.70, 0.18]	ns
Study 2b	110	(—) vs. (-)	-0.05	[-0.34, 0.24]	ns

For the pro-arguments, in contrast, the postulated significant pairwise differences can be found in three of the four studies (see Table 4). That is, in three of the four studies, there was a covariation of the evaluation bias with the polarity extremeness of the pro-arguments.

**Table 4:** Pairwise comparisons between attitudinal evaluation biases within the pro-arguments.

Group	*n*	Comparison between evaluation biases for	Estimated difference	Bayesian 99% credibility interval [lower 0.5%, upper 0.5%]	Significance
Study 1a	69	(++) vs. (+)	0.13	[0.05, 0.20]	*
Study 1b	110	(++) vs. (+)	0.08	[0.02, 0.14]	*
Study 2a	60	(++) vs. (+)	0.05	[-0.02, 0.12]	ns
Study 2b	110	(++) vs. (+)	0.10	[0.05, 0.16]	*
Study 1a	69	(+++) vs. (+)	0.32	[0.13, 0.51]	*
Study 1b	110	(+++) vs. (+)	0.19	[0.04, 0.34]	*
Study 2a	60	(+++) vs. (+)	0.10	[-0.04, 0.24]	ns
Study 2b	110	(+++) vs. (+)	0.20	[0.10, 0.31]	*
Study 1a	69	(+++) vs. (++)	0.19	[0.08, 0.30]	*
Study 1b	110	(+++) vs. (++)	0.11	[0.02, 0.20]	*
Study 2a	60	(+++) vs. (++)	0.05	[-0.02, 0.12]	ns
Study 2b	110	(+++) vs. (++)	0.10	[0.05, 0.16]	*

* Bayesian 99% credibility interval does not contain the value of zero (significant). ns: Bayesian 99% credibility interval contains the value of zero (not significant).

Altogether, the hypotheses H-3a, H-3b, and H-3c about evaluation bias differences between the three con-arguments must be rejected in all four studies. These results go along with the findings that the influence of attitude on the within-level slope for the con-arguments (first part of individual trajectories) was not significant in any of our studies. In contrast, the hypotheses H-3d, H-3e, and H-3f about bias magnitude differences between the three pro-arguments can be maintained in at least three of our four studies. Thus, an appropriate final conclusion seems to be that the evaluation bias magnitude co-varies at the polarity range of the pro-arguments (with higher magnitudes for higher polarity values), whereas it seems to be rather constant, or at least without any systematic pattern, over the whole polarity range of the con-arguments.

## Discussion and Conclusions

The aim of our study was to explore the evaluation bias over the whole range of pro- and con-arguments, using M-learning and MOOCs as topics for stimulating reactions. For this purpose, the application of (bi-linear) piecewise growth curve modeling seems to be a successful approach. Moreover, in three of the four studied groups it was more appropriate than using a simple growth curve approach with only one within-level slope for both kinds of arguments. Additionally, this more sophisticated approach allowed for separate inspection of the polarity ranges of pro- and con-arguments.

The results reveal that there were no significant effects of attitude on the within-level slopes for the con-arguments (the first part of the individual trajectories). However, in three of the four groups, significant effects of attitude on the within-level slopes for the pro-arguments appeared (i.e., for the second part of the individual trajectories).

Inspection of the evaluation bias (the assigning of higher ratings to attitude-consistent and lower ratings to attitude-inconsistent arguments) revealed significant positive attitudinal bias effects on the evaluation of all three pro-arguments in both groups and for both topics. In contrast, the results for the con-arguments were mixed and less clear. Although mostly negative in its sign, from all evaluation biases that could be estimated for each group, topic, and for each con-argument, only a quarter of these estimates reached significance. Pairwise comparisons of the evaluation biases within the con- and pro-arguments showed that the magnitude of the evaluation bias for the pro-arguments differed between them in three of the four groups. This result resembles the finding that in (the same) three of the four groups a significant effect of attitude on the within-slopes for the pro-arguments were also found. In contrast, no significant bias magnitude differences could be found between the con-arguments.

Altogether, the attitudinal evaluation bias varied in its magnitude within the pro-attitude polarity range (with higher biased ratings at more extreme pro-arguments), whereas the evaluation bias seemed to be stable (and/or weak to almost non-existent) for the con-arguments. This observed phenomenon describes an *asymmetry* in the drift of the evaluation bias over the polarity range of con- and pro-arguments.

This asymmetry did not appear within Study 2a, however. The sample in Study 2a was characterized by comparably high favorable attitudes toward M-learning. It is possible that these favorable attitudes resulted in high magnitudes of evaluation bias for a large range of pro-arguments’ polarity, and that there was little space for the evaluation bias to vary in its (high) magnitude from one pro-argument to another. Moreover, it is possible that the people in Study 2a, characterized by relatively high self-rated knowledge as well as by a high favorable attitude toward M-learning, subjectively saw only little polarity differences between the three pro-arguments and, as a consequence, they did not differentiate enough between them during the assessment of the pro-arguments.

Regarding external validity, it seems premature to come to the general conclusion that the evaluation bias varies in its magnitude primarily for pro-arguments, even though the question arises as to why such a bias drift was not observable for the con-arguments. It could be that the evaluation of con-arguments required more cognitive effort in general and, as a consequence, there was less space for an evaluation bias to vary with the polarity. Another possibility is that most participants did not realize the con-arguments’ polarity or that they did not take these polarity differences into account. Thus, in comparison to pro-arguments, con-arguments were seemingly not differentiated enough with regard to their polarity. It is also possible that the con-arguments did not sufficiently cover the whole range of the polarity scale, although care was taken in the pilot studies to choose only arguments with similar persuasiveness scores and for which the expert ratings resulted in high inter-rater agreement.

For each of these possible explanations, domain- and population-dependencies must be considered. The MOOC and the M-learning topic are future-oriented and rather positively valued issues. Hence, in future research, not only other issues but especially more controversial or emotionally charged topics should be examined (e.g., political, religious, or ethnic conflicts). At the moment, we cannot rule out that the asymmetry will eventually disappear for more negative-valued topics. Moreover, it is even imaginable that the direction of the asymmetry might be reversed for more negatively valued topics.

In any case, attention should be paid to the fact that the significance of any issue is always dependent upon its meaning to the particular subpopulation which is studied. The groups in our Studies 1a and 2a had a higher self-rated knowledge than the groups in Studies 1b and 2b, and with regard to the M-learning topic, the group in Study 2a had a comparatively more favorable attitude than the group in Study 2b. Additionally, the proportion of women was much lower and the mean age was much higher in Studies 1a and 2a than in the Studies 2a and 2b. However, even if the group membership was confounded with many third variables, disentangling these effects is out of the scope of the present research, but it should be considered in future studies. Nevertheless, with the samples used in the present studies, and especially with regard to the e-teaching community, a much higher degree of ecological validity and potential reproducibility was reached than is possible by using only a homogeneous group of university students dealing with an artificial issue (e.g., see [62]). With regard to the comparatively high self-rated knowledge of the participants in Studies 1a and 2a, it seems clear that even real domain-experts are not immune to evaluation bias in their own domain [63–65]. Taken together, future research should explore for which topics and communities certain effects can be found.

An important limitation of our study has to be noted with regard to the dependent variable. Although the arguments’ polarity scores were indeed mean values (averaged over the raters in the pilot studies; e.g., see [66]), the individual persuasiveness ratings of the arguments in the main studies were measured with a single-item rating scale with six ordered categories. The question as to whether and when such rating scales can be treated as interval-scaled variables with regard to the analysis method is an issue which is hotly debated between “purists” and “pragmatics” ([67], p. 181). Treating rating scales as quasi-interval scaled continuous variables seems to be appropriate for the investigation of new phenomena, if that approach results in consistent and important theoretical insights, and if that approach delivers similar findings to those of more sophisticated methods for categorical outcomes ([67], p. 182; see also [68–69]).

In this sense, single-item rating scales were successfully used, for example, to measure the subjective convincingness of presented material (e.g., a scale ranging from *completely unconvincing* to *completely convincing*; [11], p. 2101), to measure the participant’s self-rated political ideology (e.g., a scale ranging from *extremely left* to *extremely right*; [70], p. 1428; see [71–72]) or to assess the physical attractiveness of target persons (e.g., a scale ranging from *not attractive* to *very attractive*; [43], p. 203; see [42]). The single-item persuasiveness rating-scale in our studies, in which participants should read and judge arguments in an ecologically valid way, was used in the same sense methodologically. Additionally, it must be emphasized that in rare cases, the same argument was opened and rated more than once by the same participant. Such multiple evaluations of the same argument were averaged and accordingly, the corresponding persuasiveness ratings could take on values other than 1, 2, 3, 4, 5, and 6.

However, future studies should replicate our result patterns with persuasiveness scales that consist of a set of several items or by adapting our basic ideas to approaches for categorical outcomes (e.g., [34, 73]). Nevertheless, the fact that we could find similar results in four independent and different samples could be taken as an indication that our findings are indeed substantial and not mere methodological artifacts.

Finally, the usage of the Bayesian estimator provided some advantages; for example, the avoidance of parameter estimates with implausible values, which can result with Maximum-Likelihood estimators [46]. In future research, other advantages of Bayesian methods should be taken into consideration (e.g., the usage of informative instead of non-informative priors; see [46–47]). Additionally, the usage of (two-part) piecewise growth curve modeling seems to be more appropriate than (a) the simple dichotomizing of arguments into two categories and more appropriate than (b) the usage of simple (single-part) growth curve models with only one within-level slope for all arguments. With more arguments, more complex models can allow for more specifications and be tested (e.g., a three-part piecewise growth curve model; see [35, 37]).

## Supporting Information

S1 Table Group-specific between-level parameters for the prediction of the within-level intercept π_0i_. (PDF)

S2 Table Group-specific between-level parameters for the prediction of the within-level slope π_1i_. (PDF)

S3 Table Group-specific between-level parameters for the prediction of the within-level slope π_2i_. (PDF)

S4 Table Group-specific attitudinal evaluation bias for each con-argument. (PDF)

S5 Table Group-specific attitudinal evaluation bias for each pro-argument. (PDF)

S1 File Data for the main analyses. (DAT)

S2 File Mplus syntax. (TXT)
