# Supplementary material for: A New Method for Re-Analyzing Evaluation Bias: Piecewise Growth Curve Modeling Reveals an Asymmetry in the Evaluation of Pro and Con Arguments
Source: PLoS One. 2016 Feb 3;11(2):e0148283. doi: 10.1371/journal.pone.0148283 (PMC4739729; doi:10.1371/journal.pone.0148283)
Supplement: S1 Table — (PDF) [file pone.0148283.s001.pdf]

S1 Table. Group-specific between-level parameters for the prediction of the within-level intercept π_{0i}.

Topic	Group	*n*	Between-level parameter	Estimate	Bayesian 99% credibility interval [lower 0.5%, upper 0.5%]	Significance
MOOCs	Study 1a	69	Intercept β_{00}	3.56	[3.07, 4.05]	*
	Study 1b	110	Intercept β_{00}	4.20	[3.82, 4.58]	*
M-learning	Study 2a	60	Intercept β_{00}	4.28	[3.51, 5.07]	*
	Study 2b	110	Intercept β_{00}	3.89	[3.54, 4.23]	*
MOOCs	Study 1a	69	Slope β_{01}	-0.24	[-0.62, 0.14]	ns
	Study 1b	110	Slope β_{01}	-0.25	[-0.59, 0.11]	ns
M-learning	Study 2a	60	Slope β_{01}	-0.38	[-0.86, 0.10]	ns†
	Study 2b	110	Slope β_{01}	-0.34	[-0.64, -0.04]	*

* Bayesian 99% credibility interval does not contain the value of zero (significant).
ns: Bayesian 99% credibility interval contains the value of zero (not significant).
† A 95% credibility interval would not contain the value of zero.
