# Supplementary material for: A New Method for Re-Analyzing Evaluation Bias: Piecewise Growth Curve Modeling Reveals an Asymmetry in the Evaluation of Pro and Con Arguments
Source: PLoS One. 2016 Feb 3;11(2):e0148283. doi: 10.1371/journal.pone.0148283 (PMC4739729; doi:10.1371/journal.pone.0148283)
Supplement: S2 Table — (PDF) [file pone.0148283.s002.pdf]

S2 Table. Group-specific between-level parameters for the prediction of the within-level slope π_{1i}.

Topic	Group	*n*	Between-level parameter	Estimate	Bayesian 99% credibility interval [lower 0.5%, upper 0.5%]	Significance
MOOCs	Study 1a	69	Intercept β_{10}	0.07	[-0.53, 0.72]	ns
	Study 1b	110	Intercept β_{10}	-0.20	[-0.61, 0.23]	ns
M-learning	Study 2a	60	Intercept β_{10}	0.87	[-0.93, 2.57]	ns
	Study 2b	110	Intercept β_{10}	0.92	[0.10, 1.69]	*
MOOCs	Study 1a	69	Slope β_{11}	-0.09	[-0.57, 0.37]	ns
	Study 1b	110	Slope β_{11}	0.13	[-0.24, 0.51]	ns
M-learning	Study 2a	60	Slope β_{11}	0.62	[-0.42, 1.68]	ns
	Study 2b	110	Slope β_{11}	0.12	[-0.59, 0.82]	ns

* Bayesian 99% credibility interval does not contain the value of zero (significant).
ns: Bayesian 99% credibility interval contains the value of zero (not significant).
