# Supplementary material for: A New Method for Re-Analyzing Evaluation Bias: Piecewise Growth Curve Modeling Reveals an Asymmetry in the Evaluation of Pro and Con Arguments
Source: PLoS One. 2016 Feb 3;11(2):e0148283. doi: 10.1371/journal.pone.0148283 (PMC4739729; doi:10.1371/journal.pone.0148283)
Supplement: S3 Table — (PDF) [file pone.0148283.s003.pdf]

S3 Table. Group-specific between-level parameters for the prediction of the within-level slope π_{2i}.

Topic	Group	*n*	Between-level parameter	Estimate	Bayesian 99% credibility interval [lower 0.5%, upper 0.5%]	Significance
MOOCs	Study 1a	69	Intercept β_{20}	0.22	[-0.17, 0.57]	ns
	Study 1b	110	Intercept β_{20}	0.00	[-0.24, 0.22]	ns
M-learning	Study 2a	60	Intercept β_{20}	-0.05	[-0.38, 0.28]	ns
	Study 2b	110	Intercept β_{20}	-0.12	[-0.30, 0.07]	ns
MOOCs	Study 1a	69	Slope β_{21}	0.44	[0.18, 0.71]	*
	Study 1b	110	Slope β_{21}	0.26	[0.06, 0.47]	*
M-learning	Study 2a	60	Slope β_{21}	0.15	[-0.05, 0.35]	ns
	Study 2b	110	Slope β_{21}	0.31	[0.15, 0.47]	*

* Bayesian 99% credibility interval does not contain the value of zero (significant).
ns: Bayesian 99% credibility interval contains the value of zero (not significant).
