# Supplementary material for: A New Method for Re-Analyzing Evaluation Bias: Piecewise Growth Curve Modeling Reveals an Asymmetry in the Evaluation of Pro and Con Arguments
Source: PLoS One. 2016 Feb 3;11(2):e0148283. doi: 10.1371/journal.pone.0148283 (PMC4739729; doi:10.1371/journal.pone.0148283)
Supplement: S4 Table — (PDF) [file pone.0148283.s004.pdf]

S4 Table. Group-specific attitudinal evaluation bias for each con-argument.

Group	*n*	Argument	Estimate	Bayesian 99% credibility interval [lower 0.5%, upper 0.5%]	Significance
Study 1a	69	--- (strong con)	-0.24	[-0.62, 0.14]	ns
Study 1b	110	--- (strong con)	-0.25	[-0.59, 0.11]	ns
Study 2a	60	--- (strong con)	-0.38	[-0.86, 0.10]	ns†
Study 2b	110	--- (strong con)	-0.34	[-0.64, -0.04]	*
Study 1a	69	-- (moderate con)	-0.28	[-0.55, -0.01]	*
Study 1b	110	-- (moderate con)	-0.18	[-0.43, 0.07]	ns
Study 2a	60	-- (moderate con)	-0.24	[-0.60, 0.11]	ns
Study 2b	110	-- (moderate con)	-0.31	[-0.53, -0.10]	*
Study 1a	69	- (weak con)	-0.36	[-0.80, 0.06]	ns†
Study 1b	110	- (weak con)	-0.07	[-0.41, 0.26]	ns
Study 2a	60	- (weak con)	0.02	[-0.46, 0.51]	ns
Study 2b	110	- (weak con)	-0.26	[-0.58, 0.05]	ns†

* Bayesian 99% credibility interval does not contain the value of zero (significant).
ns: Bayesian 99% credibility interval contains the value of zero (not significant).
† A 95% credibility interval would not contain the value of zero.
