# Supplementary material for: A New Method for Re-Analyzing Evaluation Bias: Piecewise Growth Curve Modeling Reveals an Asymmetry in the Evaluation of Pro and Con Arguments
Source: PLoS One. 2016 Feb 3;11(2):e0148283. doi: 10.1371/journal.pone.0148283 (PMC4739729; doi:10.1371/journal.pone.0148283)
Supplement: S5 Table — (PDF) [file pone.0148283.s005.pdf]

S5 Table. Group-specific attitudinal evaluation bias for each pro-argument.

Group	*n*	Argument	Estimate	Bayesian 99% credibility interval [lower 0.5%, upper 0.5%]	Significance
Study 1a	69	+ (weak pro)	0.26	[0.04, 0.48]	*
Study 1b	110	+ (weak pro)	0.30	[0.11, 0.48]	*
Study 2a	60	+ (weak pro)	0.31	[0.09, 0.53]	*
Study 2b	110	+ (weak pro)	0.32	[0.14, 0.51]	*
Study 1a	69	++ (moderate pro)	0.39	[0.16, 0.62]	*
Study 1b	110	++ (moderate pro)	0.37	[0.17, 0.57]	*
Study 2a	60	++ (moderate pro)	0.36	[0.14, 0.57]	*
Study 2b	110	++ (moderate pro)	0.43	[0.22, 0.63]	*
Study 1a	69	+++ (strong pro)	0.58	[0.29, 0.87]	*
Study 1b	110	+++ (strong pro)	0.48	[0.24, 0.73]	*
Study 2a	60	+++ (strong pro)	0.41	[0.18, 0.63]	*
Study 2b	110	+++ (strong pro)	0.53	[0.29, 0.76]	*

* Bayesian 99% credibility interval does not contain the value of zero (significant).
